# Alzheimer’s disease after mild traumatic brain injury

**DOI:** 10.18632/aging.204179

**Published:** 2022-07-12

**Authors:** Phoebe Imms, Helena C. Chui, Andrei Irimia

**Affiliations:** 1 Leonard Davis School of Gerontology, University of Southern California, Los Angeles, CA 90089, USA; 2 Department of Neurology, Keck School of Medicine, University of Southern California, Los Angeles, CA 90033 USA; 3 Alzheimer’s Disease Research Center, Keck School of Medicine, University of Southern California, Los Angeles, CA 90033, USA; 4 Corwin D. Denney Research Center, Department of Biomedical Engineering, Viterbi School of Engineering, University of Southern California, Los Angeles, CA 90089, USA

**Keywords:** dementia, traumatic brain injury, magnetic resonance imaging, prognosis, machine learning

Traumatic brain injury (TBI) is associated with cognitive, somatomotor, and behavioral dysfunction that may persist indefinitely [1] and, in some cases, with increased risk of progressive cognitive impairment and dementia, often diagnosed as Alzheimer disease (AD) or chronic traumatic encephalopathy. TBI is associated with brain accumulation of amyloid β protein and τ neurofibrillary tangles (both of which are considered pathological hallmarks of AD), as well as with acute cerebral microbleeds that might initiate or aggravate neurodegenerative processes in older adults [2]. Although TBI is associated with increased risk of incident AD, the nature of and interactions between TBI and neurodegeneration are unclear and the early identification of TBI patients at risk for onset of AD pathology and progressive cognitive decline remains challenging.

Mild TBI (mTBI), which accounts for ~75% of all TBIs, exhibits considerable heterogeneity pertaining to risk of progressive cognitive impairment and AD pathology. Early post-TBI assessment of risk for progressive cognitive decline could inform clinical monitoring, cognitive training and/or other therapeutic interventions. With the recent US Food and Drug Administration approval of aducanumab and with other disease-modifying treatments directed at amyloid and τ that are undergoing clinical trials, this research topic is of considerable interest to federal research agencies, which offer funding initiatives aimed at explicating AD risk after TBI.

We have proposed, developed, and tested approaches that combine structural and functional neuroimaging with machine learning to identify persons with mTBI who are at risk for progressive cognitive decline. Structural and functional brain dynamics after TBI are paralleled by older-than-expected (biological) brain ages, which can be estimated using multivariate models of neuroanatomical features derived from magnetic resonance imaging (MRI) [3]. Structurally, the severity of brain atrophy (described as older-than-expected brain age) is strongly predictive of dementia risk. Both TBI- and AD-related neurodegeneration can exhibit cortical thinning patterns that are statistically indistinguishable across these conditions [4]. Furthermore, both TBI and AD evince changes in the fractional anisotropy of water diffusion along white matter fasciculi connecting the lateral temporal lobe and the hippocampus [5]. By comparing changes in gray and white matter properties across mTBI and AD, we have pioneered methods for the early identification of mTBI victims with AD-like neurodegeneration patterns that may predict progressive cognitive decline [4–6]. According to our findings [4,6], early post-traumatic cognitive deficits can predict subsequent AD-like abnormalities in brain structure and function approximately six months after injury [4]. For these and similar reasons, quantitative assessment of post-traumatic changes in brain structure and function may better predict the risk of subsequent cognitive decline than cognitive assessment alone. Longer longitudinal follow-up is needed, however, to determine whether structural and functional changes at about six months post-injury represent delayed manifestations of acute TBI, the beginning of progressive cognitive decline, or both.

From a functional point of view, we have used machine learning to predict network changes based on acute cognitive deficits [6]. We found that certain patterns of performance on Montreal Cognitive Assessments obtained within a week after mTBI are predictive of alterations in the default mode network, with sensitivities and specificities exceeding 90% [7]. These findings suggest that post-traumatic patterns of neural degradation similar to those seen in AD could be forecast using standard clinical assessments obtained during the acute stage of injury. This research showcases the potential to early identify mTBI patients at risk for patterns of brain alteration that resemble those observed in AD. Given the potential translational significance of such forecasting, our findings suggest that these approaches, which synergize neuroimage analysis with cognitive assessments, should be tested for their ability to predict cognitive trajectories and pathology after mTBI.
